# NMR Characterization of an Oligonucleotide Model of the MiR-21 Pre-Element

**DOI:** 10.1371/journal.pone.0108231

**Published:** 2014-09-24

**Authors:** Sara Chirayil, Qiong Wu, Carlos Amezcua, Kevin J. Luebke

**Affiliations:** 1 Division of Cardiology, Department of Internal Medicine, University of Texas Southwestern Medical Center, Dallas, Texas, United States of America; 2 Department of Biophysics, University of Texas Southwestern Medical Center, Dallas, Texas, United States of America; 3 Structural Elucidation Group, Medical Products Division, Baxter Healthcare, Round Lake, Illinois, United States of America; University of Pittsburgh School of Medicine, United States of America

## Abstract

We have used NMR spectroscopy to characterize an oligonucleotide stem loop structure based on the pre-element of an oncogenic microRNA, miR-21. This predicted stem-loop structure is cleaved from the precursor of miR-21 (pre-miR-21) by the nuclease Dicer. It is also a critical feature recognized by the protein complex that converts the primary transcript (pri-miR-21) into the pre-miRNA. The secondary structure of the native sequence is poorly defined by NMR due to rapid exchange of imino protons with solvent; however, replacement of two adjacent putative G•U base pairs with G•C base pairs retains the conformation of the hairpin observed by chemical probing and stabilizes it sufficiently to observe most of the imino proton resonances of the molecule. The observed resonances are consistent with the predicted secondary structure. In addition, a peak due to a loop uridine suggests an interaction between it and a bulged uridine in the stem. Assignment of non-exchangeable proton resonances and characterization of NOEs and coupling constants allows inference of the following features of the structure: extrahelicity of a bulged adenosine, deviation from A-form geometry in a base-paired stem, and consecutive stacking of the adenosines in the 5′ side of the loop, the guanosine of the closing base pair, and a cross-strand adenosine. Modeling of the structure by restrained molecular dynamics suggests a basis for the interaction between the loop uridine, the bulged uridine in the stem, and an A•U base pair in the stem.

## Introduction

MicroRNAs (miRNAs) are short, non-coding RNAs that regulate gene expression by diminishing translation of their target messenger RNAs [1], [2]. Whereas their normal function is regulation of development and cellular responses to stress [3], the aberrant expression of specific miRNAs is associated with a wide range of diseases, including cancer [4] and heart disease [5], [6]. For example, miR-21 is a miRNA that is elevated in both cancer and heart disease. It is highly expressed in a variety of tumors [7], contributing to the cancer phenotype by diminishing translation of tumor suppressor genes [8]–[12]. It is also expressed in hypertrophic heart tissue, where it contributes to the fibrotic response to cardiac stress or injury [13]. An understanding of the factors that regulate miRNA expression is essential to efforts to therapeutically target specific disease-related miRNAs [14], [15] and to gaining a basic understanding of the roles of miRNAs in biology.

**Figure 1:**
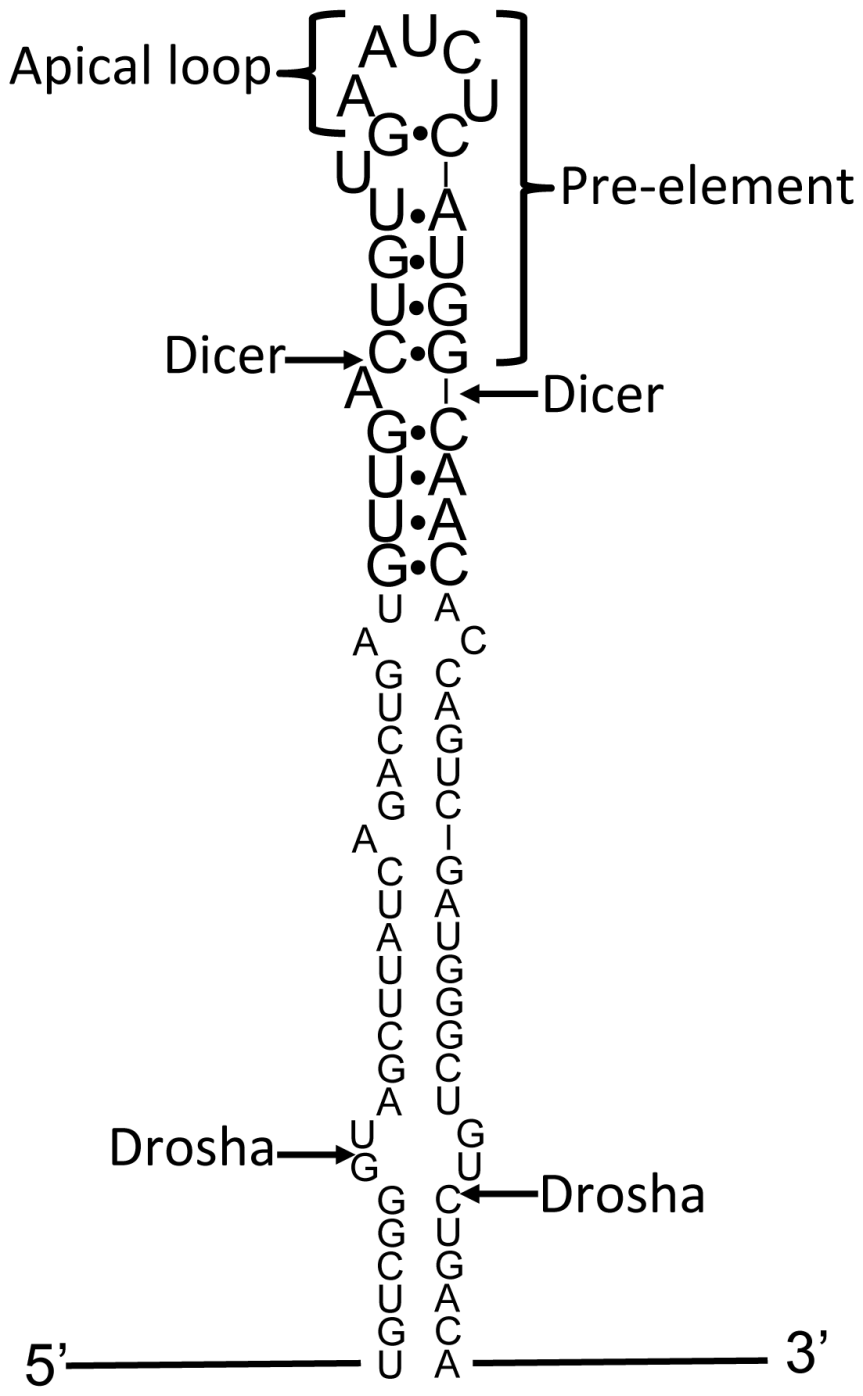
Structural features of pri-miRNAs. The stem and apical loop sequences of pri-miR-21 are shown. Arrows indicate sites of cleavage by Drosha and Dicer, as indicated. The sequence of nucleotides shown in large font is investigated in this work.

Expression of miRNAs is regulated post-transcriptionally by modulation of their maturation [16], [17]. MiRNAs are initially transcribed within much longer RNAs (pri-miRNAs), which are subsequently processed in a series of steps to produce the mature miRNA [1] (Figure 1). The first step of this process is cleavage of a long hairpin structure, which contains the mature miRNA sequence, from the primary transcript. A multiprotein complex called the Microprocessor effects this processing step, distinguishing hairpins that contain miRNAs from the multitude of other hairpin structures in the transcriptome [18]–[22]. The excised hairpin is exported to the cytoplasm [23], [24], where the nuclease Dicer cleaves the mature miRNA from the precursor hairpin (pre-miRNA) [25] by removing a structure called the pre-element, a short stem-loop comprising the terminal loop and a short region of predicted base pairing. This structure is also known as the terminal loop region or apical region. The remaining duplex associates with an Argonaute protein, leading to the retention of the single-stranded miRNA in the active miRNA-Argonaute complex [26].

Several lines of evidence establish that the pre-element is a critical feature for defining miRNAs and regulating their production from primary transcripts. All human pri-miRNAs contain a terminal loop [1], and many pri-miRNAs have highly conserved loop sequences [27]. Mutations that decrease the size of the loop or stabilize the nominally base-paired region in the pre-elements of a number of miRNA precursors inhibit processing of pri-miRs by Drosha and pre-miRs by Dicer, suggesting that these nucleases require conformational flexibility in this region of their substrates for maximal activity [28], [29]. Furthermore, several auxiliary factors that modulate Drosha and Dicer cleavage, including hnRNP A1, Lin-28, and KSRP, regulate processing by binding to the terminal loops of specific miRNA precursors [27], [30]–[32].

Processing of miR-21 is of special interest because of its potential as a therapeutic target. Heightened expression of miR-21 in tumor cells suppresses translation of pro-apoptotic genes, allowing cancer cells to evade apoptosis [33]. MiRNA-targeting antisense agents, called antagomirs, directed to miR-21 stimulate apoptosis or increased sensitivity to pro-apoptotic drugs in tumor cells [33], [34], and genetic deletion of miR-21 in a mouse model of non-small cell lung cancer protects against tumor formation [35]. MiR-21 is also upregulated in cardiac fibroblasts in failing mouse and human hearts [5], [36], and antimiR-mediated inhibition of miR-21 attenuates fibrosis and improves cardiac function in mouse models of heart failure [13]. Thus, agents that diminish production of this miRNA hold great promise as treatments for disease as well as probes of miR-21 function.

We and others are interested in modulating the processing of miR-21 with ligands specific for its pre-element [37]–[39]. Compounds that recognize the putatively base-paired component of miRNA pre-elements can inhibit cleavage by Dicer [37], [40], [41] and compounds that bind to the terminal loops of pri-miRs can inhibit cleavage by the microprocessor [27], [42], [43]. Such compounds could also affect the association of auxiliary factors that influence the processing of specific miRNAs [27]. The terminal loop of miR-21 is highly conserved, being identical in every mammalian pri-miR-21 listed in miRBase [44], suggesting the importance of such auxiliary factors in regulation of miR-21 processing.

Development of ligands for the pre-element of miR-21 will be aided by information about its conformation. The predicted secondary structure of this RNA, shown in Figure 1, includes a five-nucleotide loop with a single bulged (i.e., unpaired) nucleotide adjacent to the closing G•C base pair. Few structural models of five-nucleotide RNA hairpin loops are available to guide prediction of the conformation of this structure. We report here characterization by NMR spectroscopy and in-line probing of an oligonucleotide model of this RNA.

## Results

### Exchangeable Protons and Secondary Structure

**Figure 2:**
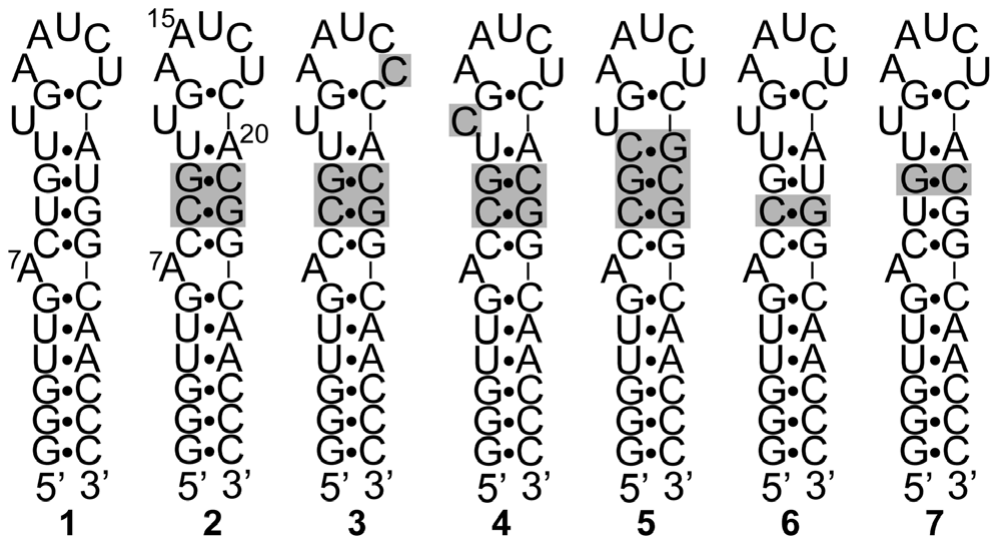
RNA sequences characterized in this work. RNA **1** corresponds to the native sequence from pri-miR-21. Variations from the sequence of **1** are highlighted by shading in RNAs **2–7**.

**Figure 3:**
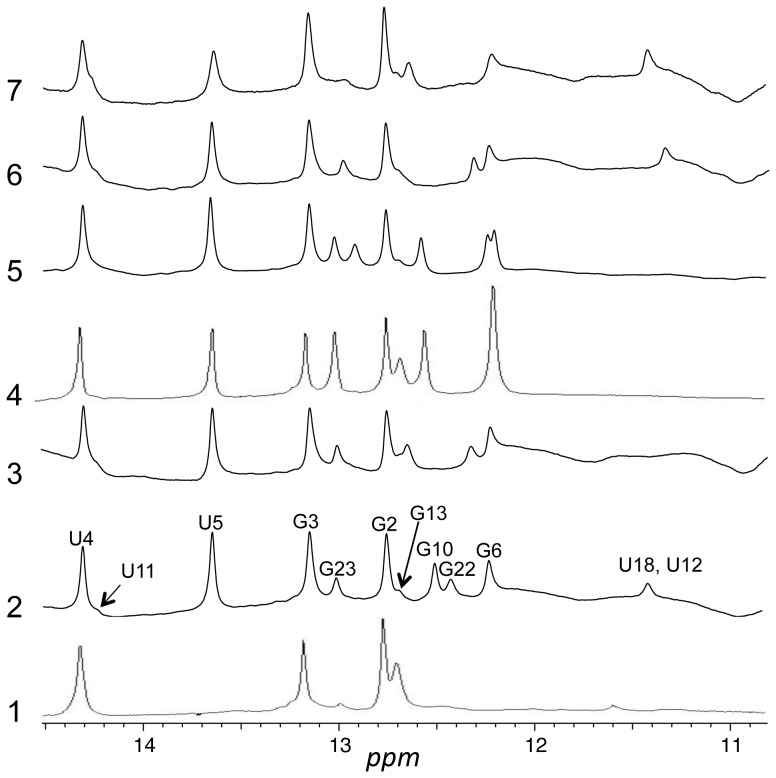
NMR spectra of imino protons for RNAs 1–7 in H_2_O. Peaks for **2** are labeled according to assignments described in the text. Conditions were as described in Materials and Methods.

We initially investigated the NMR spectrum of RNA **1** (Figure 2), which directly models the pre-element of miR-21. The bulged adenosine (A7) corresponds to the 3′-terminal nucleotide of the mature miRNA, the pre-element being the stem and loop structure above this bulge. Four base pairs immediately below the bulge correspond to the base pair sequence at that position in the precursor miRNA, and two additional G•C base pairs were added to stabilize the lower stem and provide for the possibility of in vitro transcription. We previously confirmed by UV-monitored thermal denaturation studies that this sequence folds into a unimolecular hairpin structure [38]. The imino proton region of RNA **1** in H_2_O is shown in Figure 3. Only 6 of the possible 16 imino protons in this molecule are sufficiently protected from exchange with H_2_O to be represented by peaks in the spectrum. The ready exchange with water of the majority of the imino protons suggests a dynamic or disordered structure for the RNA.

**Figure 4:**
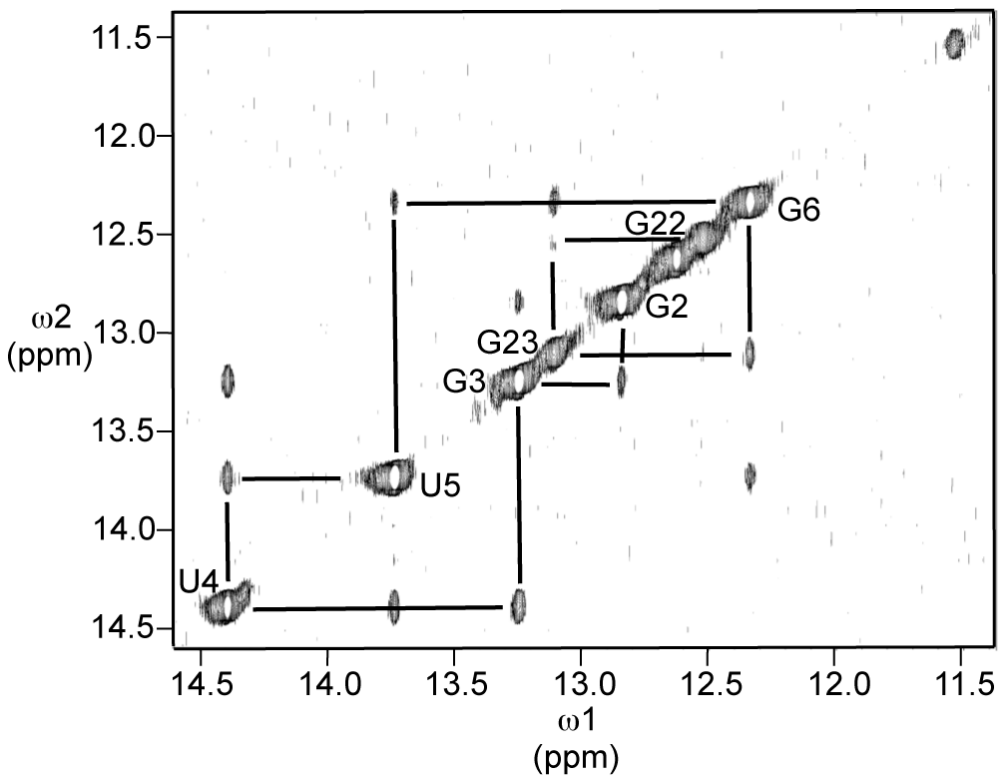
Imino proton region of NOESY spectrum of 2 in H_2_O. Conditions were as described in Materials and Methods. Sequential NOEs are indicated by lines to peaks due to proximal protons. Peaks are labeled according to assignments based on sequential NOEs.

To create a more stable conformation for structure determination, we replaced the putative G•U pairs in the upper stem with G•C base pairs in RNA **2**. The imino proton region of RNA **2** is shown in Figure 3. A more stable base paired structure is indicated by the appearance of peaks for at least 11 of the 14 imino protons in the molecule. The correspondence in chemical shifts between the imino protons that are visible in both **1** and **2** suggests that **1** has a similar conformation to **2**. Therefore, **1** is likely dynamic rather than disordered. A series of sequential NOEs in the NOESY spectrum of **2** in H_2_O (Figure 4) from G2 through G22 indicates formation of the predicted base pairs by each of these nucleotides. An NOE between the imino protons of G6 and G23 indicates continuous stacking of the base pairs formed by these nucleotides.

In addition to the sequentially proximal imino protons, four other imino protons are evident in the spectrum. A shoulder at 14.2 ppm is consistent with formation of the A•U base pair between A20 and U11. A very broad peak between 11 and 12 ppm is consistent with an unpaired uridine. Peaks at 12.7 and 11.4 ppm could indicate the presence of an additional G•C base pair (12.7 ppm) such as the predicted pair between G13 and C19 and a uridine (11.4 ppm) engaged in a non-Watson-Crick interaction. Alternatively, it could signal the presence of an unexpected G•U base pair, with the guanosine imino resonance at 11.4 ppm and the uridine imino resonance at 12.7 ppm.

The absence of an NOE between the peaks at 11.4 ppm and 12.7 ppm argues against the involvement of these two protons in a G•U base pair. Furthermore, though replacement of U18, the most likely participant in a G•U pair with G13, by a cytosine (RNA **3**) results in disappearance of the peak at 11.4 ppm, it does not result in the appearance of a new peak corresponding to a new G•C base pair. Similarly, replacement of U12 with a cytosine (RNA **4**) also eliminates the peak at 11.4 ppm as well as the broad peak from 11 ppm to 12 ppm. This replacement does not result in appearance of a new peak due to a G•C base pair either but does result in intensification of the peak at 12.7 ppm.

To confirm the assignment of the shoulder at 14.2 ppm to the base pair between A20 and U11, we replaced these nucleotides with a guanosine and cytosine, respectively (RNA **5**). As anticipated, the shoulder at 14.2 ppm is absent in the spectrum of this RNA and a new peak at 12.9 ppm, corresponding to a G•C base pair, appears. Somewhat unexpectedly, both the broad peak at 11 ppm-12 ppm and the small peak at 11.4 ppm are absent in the spectrum of **5**.

To confirm the assignments of G22 and G10 and assess the overall effect of the replacement of a G•C base pair for each individual G•U pair in the upper stem, spectra were acquired for RNAs **6** and **7**, in which C21 and C9 of **2** were replaced with uridines, respectively. As expected, peaks at 12.5 ppm and 12.4 ppm, respectively, were absent. Furthermore, these alterations had little effect on the spectra beyond those peaks.

### In-Line Probing of RNAs 1 and 2

Mg^2+^-induced hydrolytic cleavage was used to characterize and compare the conformations of RNAs **1** and **2**. Hydrolytic cleavage of the RNA backbone occurs principally through nucleophilic attack of a 2′-hydroxyl on the adjacent phosphodiester, displacing the 5′-hydroxyl of the following nucleotide. For this displacement to occur, the attacking 2′-hydroxyl must be in-line with the scissile phosphorus–oxygen bond [45]. Thus, in-line cleavage, which is stimulated by divalent metal ions such as Mg^2+^, is a useful probe of conformation and flexibility.

**Figure 5:**
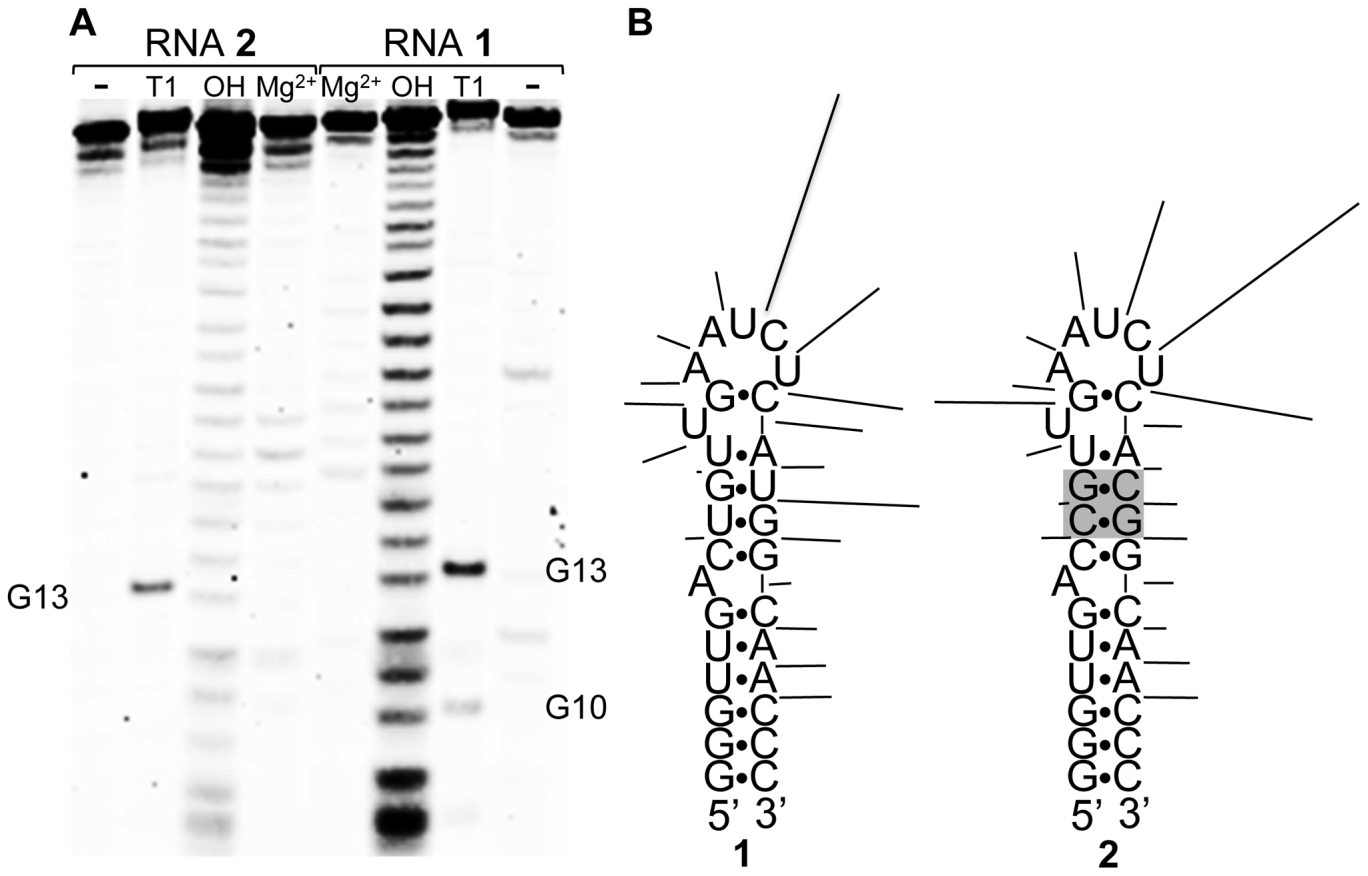
Mg^2+^-stimulated (in-line) cleavage of RNAs 1 and 2. A. Electrophoretic analysis of cleavage. Mg^2+^: RNA treated with 5 mM MgCl_2_. OH: hydrolysis ladder. T1: Ribonuclease T1 digestion. Untreated RNA in lanes labeled “-“. Bands due to guanosines identified from RNAse T1 digestion are indicated. B. Mapping of cleavage onto predicted secondary structures of **1** and **2**. Cleavage after each nucleotide is indicated by a line with length proportional to band intensity.

Electrophoretic analysis of Mg^2+^-stimulated cleavage of RNAs **1** and **2** is shown in Figure 5. The cleavage patterns are similar, with most intense cleavage occurring after the 3 nucleotides at the 3′ side of the predicted loop. Both also show cleavage along the 3′ strand of the stem. At a quantitative level, some differences in the cleavage patterns are apparent. Specifically, cleavage after U12 is stronger in **2**; whereas, cleavage after nucleotide 21 (C21 in **2**, U21 in **1**) is stronger in **1**. Also, cleavage in the loop is strongest after U16 in **1** but strongest after C17 in **2**.

### Assignment of Non-exchangeable protons of RNA 2

**Figure 6:**
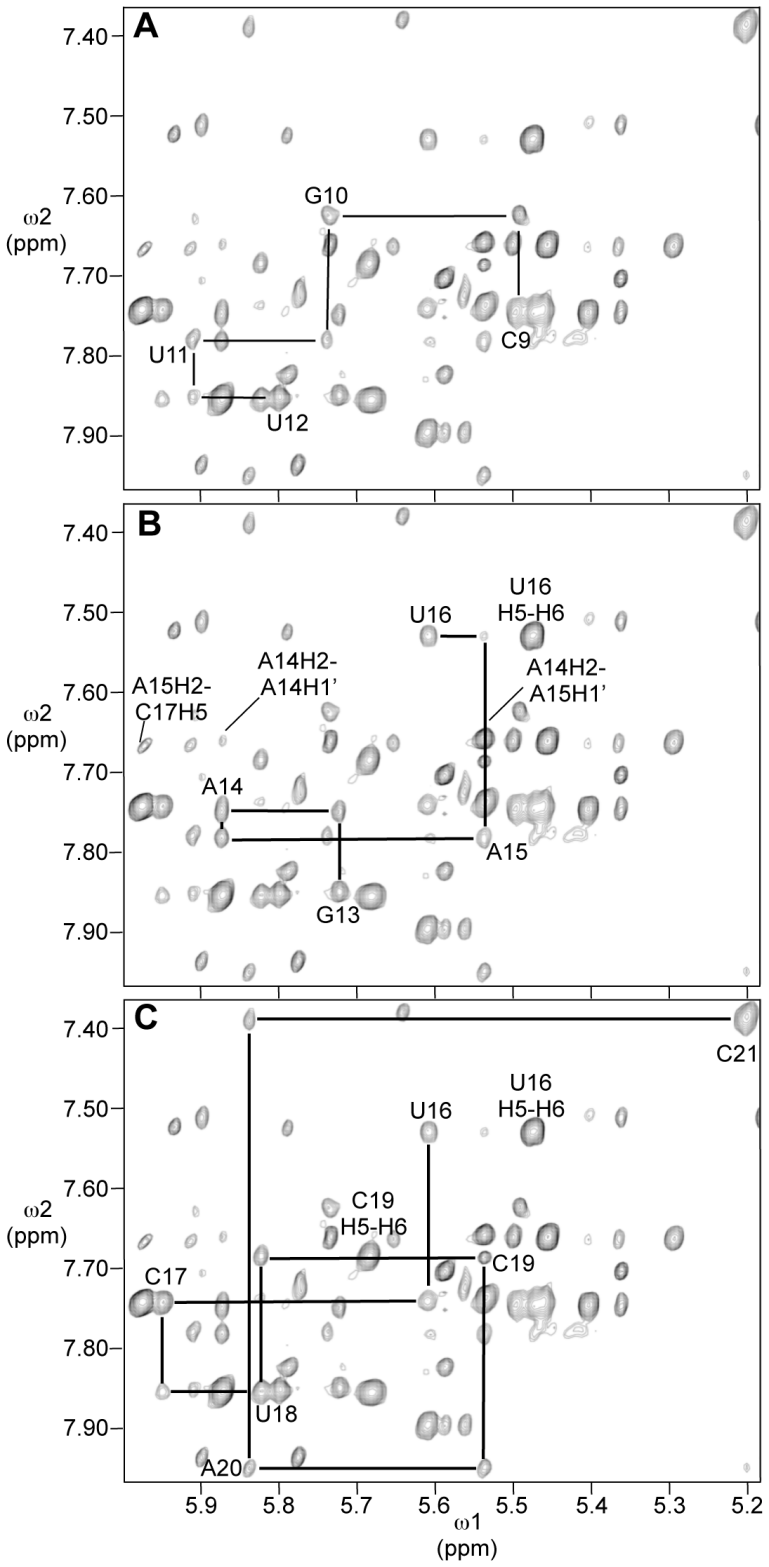
Portion of the NOESY spectrum showing NOEs between H8/H6/H2 (7.4–8.0 ppm) and H1′/H5 (5.2–6.0 ppm) protons. Mixing time was 400 ms and temperature was 25°C. Cross-peaks due to NOEs from a nucleotide aromatic proton to the H1′ proton of its own sugar are labeled. A. Sequential NOEs from C9 to U12. B. Sequential NOEs from G13 to U16. Crosspeaks due to H2 of A14 and A15 and the U16 H5 to U16 H6 are labeled. C. Sequential NOEs from U16 to C21.

Assignment of non-exchangeable protons followed standard procedures based on sequential NOE connectivities and through-bond correlations [46]. The chemical shifts of assigned non-exchangeable and exchangeable protons of RNA **2** are listed in Table 1. Pyrimidine H5 and H6 resonances were identified by their strong crosspeaks in the double-quantum filtered COSY spectrum of the molecule. Cytosines were further distinguished from uridines by the chemical shifts of their C5 carbons, determined in a natural abundance ^1^H-^13^C HSQC spectrum. NOE connectivities were observed in a 400 ms mixing-time NOESY experiment with identifiable purine-pyrimidine patterns, leading to the sequential assignment of aromatic and H1′ protons. Sequential H1′ to aromatic connectivities were nearly continuous through the molecule, broken only between C21 and G22 and broken or obscured by overlap between C8 and C9 and between U12 and G13 (Figure 6). Assignments were confirmed by sequential aromatic to aromatic and H1′ to H1′ crosspeaks.

**Table 1:** Chemical Shifts (ppm) of Assigned Protons^a^.

	H8/H6	H5/H2	H1′	H2′	H3′	H4′	imino^b^	amino^b^
G1	8.06	NA^c^	5.79	4.92	4.30	4.38		
G2	7.52	NA	5.93	4.74	4.61	4.51	12.84	8.45/6.85
G3	7.17	NA	5.77	4.54	4.39	4.49	13.25	8.36/6.97
U4	7.72	5.08	5.56	4.44	4.61	4.46	14.39	NA
U5	7.90	5.61	5.59	4.34	4.51		13.74	NA
G6	7.82	NA	5.79	4.30	4.04	4.38	12.34	8.43/6.56
A7	8.40	8.22	6.17	4.87	4.21	4.56	NA	
C8	7.73	5.56	5.45	4.24	3.98	4.16	NA	
C9	7.74	5.46	5.49	4.48	4.56	4.40	NA	
G10	7.62	NA	5.74	4.45	4.60	4.50	12.64	8.25/6.71
U11	7.78	5.41	5.91	4.41	4.71	4.46	14.34	NA
U12	7.85	5.68	5.80	4.41	4.64	4.48		NA
G13	7.85	NA	5.72	4.73		4.48	12.76	
A14	7.75	7.66	5.87	4.58	4.53	4.50	NA	
A15	7.78	7.66	5.54	4.46	4.41		NA	
U16	7.53	5.47	5.61	4.12	4.29	4.61		NA
C17	7.74	5.98	5.95	4.36	4.66	4.53	NA	
U18	7.85	5.88	5.82	4.23	4.50	4.58	11.53	NA
C19	7.69	5.68	5.54	4.62	4.30		NA	
A20	7.95	7.22	5.84	4.55	4.45	4.48	NA	
C21	7.39	5.20	5.20	4.39	4.31	4.47	NA	
G22	7.38	NA	5.64	4.42	4.62		12.52	8.13/6.67
G23	7.03	NA	5.65	4.60	4.34	4.65	13.12	8.03/6.48
C24	7.66	5.29	5.37	4.46	4.38	4.55	NA	
A25	8.05	6.78	5.78	4.46			NA	7.87/6.76
A26	7.94	7.71	5.90	4.40	4.35	4.42	NA	7.70/
C27	7.51	5.17	5.37	4.35	4.00	4.16	NA	
C28	7.75	5.41	5.51	4.24	4.00	4.34	NA	
C29	7.66	5.46	5.73	3.98	4.19	4.16	NA	8.30/6.99

a Assignments of nonexchangeable protons are at 25°C. All chemical shifts are reported relative to TSP.

b Assignments of imino and amino protons are at 10°C.

c NA: not applicable.

The 2′ protons were assigned by their crosspeaks with 1′ protons in a short mixing time (60 ms) NOESY spectrum and, for nucleotides with significant C2′-endo character, in the DQF-COSY spectrum. The 2′ assignments were confirmed by sequential H2′-H6/H8 NOE connectivities in the short mixing-time NOESY spectrum. H4′ assignments were made from the H1′-H2′/H3′/H4′/H5′/H5″ region of a NOESY spectrum with a mixing time of 150 ms, and H3′ assignments were made from the H1′-H2′/H3′/H4′/H5′/H5″ region of a NOESY spectrum with a mixing time of 400 ms.

Adenosine H2 protons were identified by the chemical shifts of bound carbons, determined in the natural abundance ^1^H-^13^C HSQC spectrum, and for adenosines 7, 20, 25, and 26, NOEs to cross-strand H1′ protons. The H2 protons of adenosines A14 and A15 had nearly identical chemical shifts, 7.66 ppm, but a small offset allowed specific assignment, based on a strong NOE between H2 of A14 and the H1′ of A15 (Figure 6B). This NOE is comparable in intensity to the intranucleotide H5-H6 NOEs and much stronger than the intranucleotide H2-H1′ NOEs for adenosines in the *anti*- conformation about the glycosidic bond, ruling out its assignment as the A15 H2-H1′ intranucleotide NOE. Further supporting this assignment, the assigned A14 H2 to A15 H1′ NOE overlaps with an NOE between the same H2 proton and C19 H1′. This overlap was resolved in a NOESY spectrum taken at 15°C (data not shown) and is most consistent with an NOE between A14H2 and C19H1′.

### Conformational Features of RNA 2

The NMR data for the lower stem of **2** (nucleotides 1–6 and 24–29) are consistent with a Watson-Crick base paired A-form double helix as anticipated. The observation of imino proton peaks with a continuous sequence of NOEs between them for these residues in addition to standard internucleotide NOEs between the nonexchangeable protons indicate a typical RNA duplex. The NOE between the imino protons of G6 and G23, without interruption by A7, indicates that the bulged adenosine is not stacked into the helix. The internucleotide NOEs between G6 and A7 and between A7 and C8 are very weak, supporting this view. However, a very weak cross-strand NOE between A7 H2 and C24 H1′ suggests that the purine heterocycle of A7 is partially associated with a groove of the duplex.

Crosspeaks between H1′ and H2′ in the DQF-COSY spectrum indicate a significant C2′-*endo* character for 13 of the sugars in the molecule. The 1′-2′ scalar couplings for these sugars and an estimate of the equilibrium percentage of each in the C3′-*endo* conformation [47] are listed in Table 2. These sugars indicate points of backbone flexibility or regions where the backbone spans a greater distance than in A-form double-helical structure. In addition to the terminal nucleotides (G1 and C29) and the bulged adenosine (A7) and preceding nucleotide (G6), many of the loop and adjacent nucleotides display C2′-*endo* character. Of the five nucleotides formally included in the loop, only A14 does not show significant C2′-*endo* character. A20 shows a large C2′-*endo* character, consistent with the requirement that it span a bulged uridine. Though G10, G13, and U11 or U12 are also apparently base paired, as indicated by the observation of a peak due to an imino proton for each, their adoption of partial C2′-*endo* character indicates that they are distorted from a canonical duplex.

**Table 2:** Summary of Ribose Sugar Conformations.

nucleotide^a^	J_1′-2′_ (Hz)	%C3′-*endo* (±10%)^b^
G1	4.8	45
G6	6.4	21
A7	6.5	21
G10	5.1	41
U11	5.9	29
U12	6.2	26
G13	4.7	47
A15	5.6	33
U16	6.7	18
C17	7.2	11
U18	2.8	75
A20	6.4	21
C29	4.6	49

a Nucleotides not included in the table are >90% in the C3′-*endo* conformation.

b Values were calculated from J_1′-2′_ using the empirical equation of van den Hoogen: %C3′-*endo*  = 114.9-14.5(J_1′-2′_) [47].

Structure predictions indicate that A20 base pairs with U11, leaving U12 unpaired, but the NMR data do not clearly distinguish between that possibility and the alternative of A20 base pairing with U12. Overlap between U12 H6 and G13 H8 resonances obscures the presence or absence of an NOE between those protons. Typical sequential internucleotide NOEs, (H8/H6-H8/H6 and H1′-H1′) are weak or not seen from G10-G13, consistent with either possibility. The in-line cleavage data, however, suggest the greatest propensity for an unpaired conformation around U12. The most effective hydrolytic cleavage flanks that nucleotide, especially following it between U12 and G13. This pattern is observed for RNA **1** and RNA **2**.

Continuous sequential NOEs from G13 through A15 suggest continuous stacking of those bases. The relatively weak Mg^2+^-induced cleavage between those nucleotides further supports that view. An NOE between A15 H1′ and U16 H6 is observed, but relatively weak (Figure 6B), and no NOE is observed between U16 H6 and C17 H6. These facts taken together suggest that the backbone trajectory turns at U16. The NOE between A15H2 and C17H5 (Figure 6B) suggests that C17 is oriented toward the inside of the loop, facing across it toward the stacked adenosines.

The dependence of the appearance of the imino proton resonance at 11.4 ppm on U18 and U12, as well as the A20•U11 base pair indicates an interaction between these nucleotides. The interaction could be direct, such as through hydrogen bonding between the interacting nucleotides, or indirect, such as a structural perturbation that affects spatially remote nucleotides.

### Structure modeling by restrained molecular dynamics

Nucleotides 8–23 were modeled using 80 torsion angle restraints, 39 distance restraints to constrain the experimentally determined base pairs (C8•G23, C9•G22, G10•C21, U11•A20, and G13•C19) to appropriate hydrogen bonding distances and base pair planarity, 29 NOE-derived intranucleotide distance restraints, 87 NOE-derived internucleotide restraints, and 100 distance restraints to model the two terminal base pairs as A-form duplex. Eighty structures were calculated and the fourteen lowest energy output structures were analyzed.

**Figure 7:**
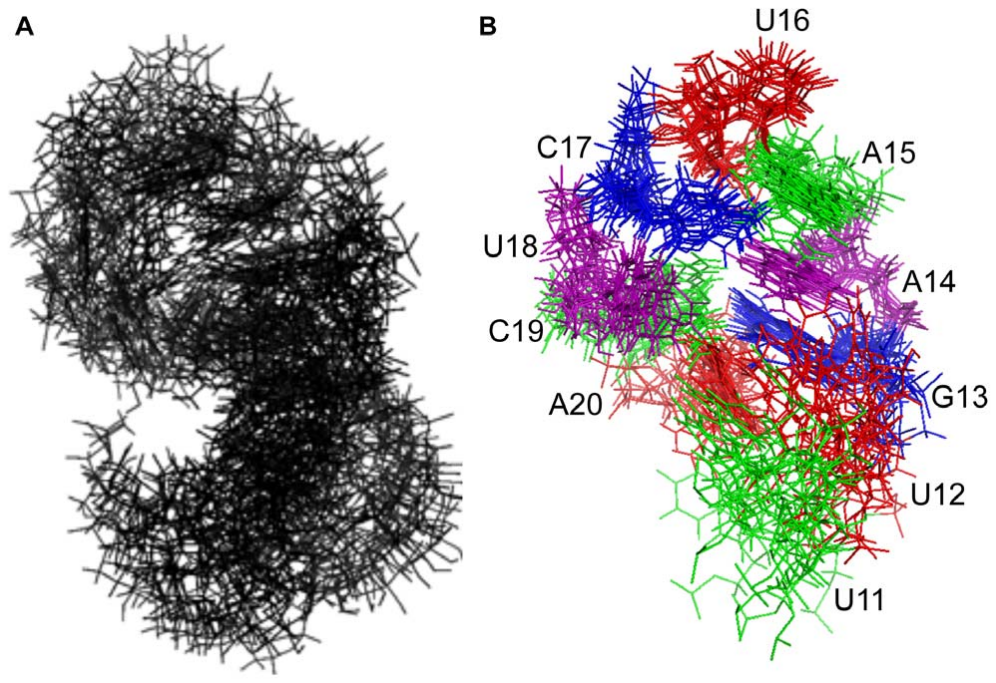
Alignment of the 14 lowest energy output structures for 2 (PDB ID 2MNC). A. Alignment of 16 nucleotides (C8–G23) included in restrained molecular dynamics calculation. B. Alignment of loop nucleotides U11 to A20. Only the aligned nucleotides are shown.

**Figure 8:**
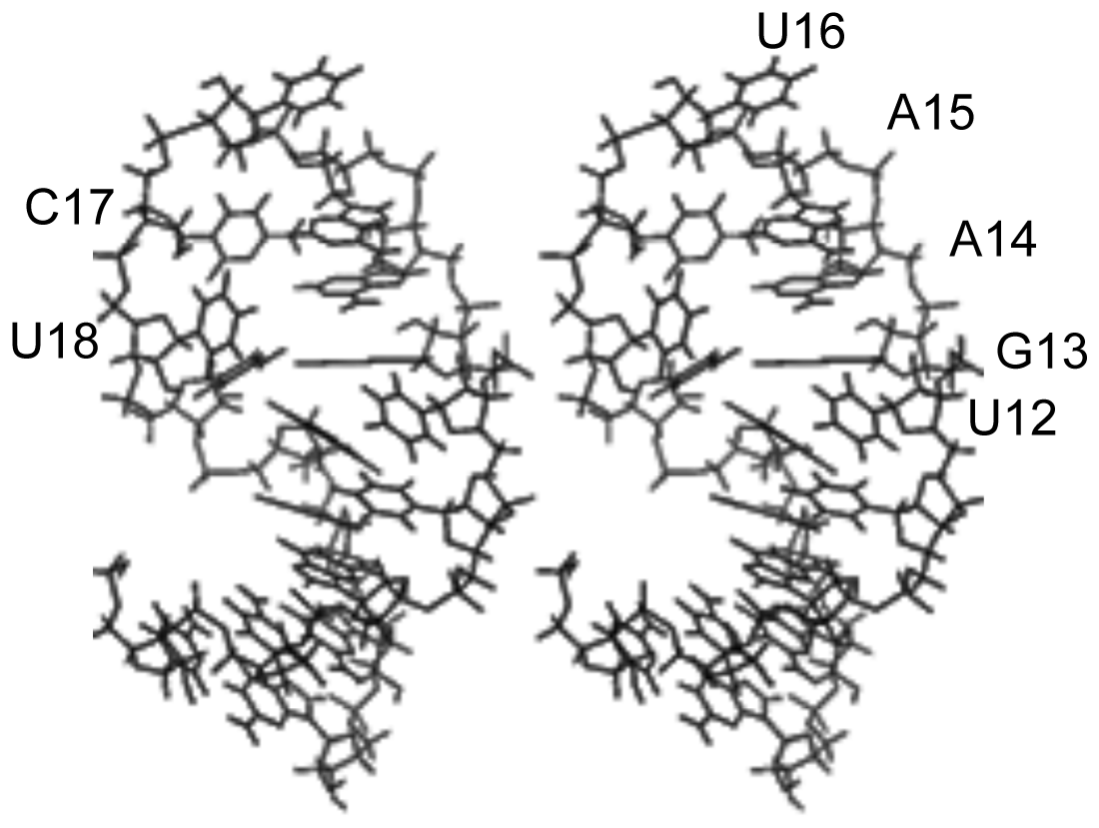
Stereoview of a representative structure for nucleotides 8–23 of RNA 2 (PDB ID 2MNC).

An alignment of the fourteen structures is shown in Figure 7A (BMRB accession code: 19887; PDB ID: 2MNC); however, the orientation of the base paired stem (lower three base pairs) with respect to the loop is poorly defined, and separate alignment of loop nucleotides 11–20 (Figure 7B) provides a clearer view of the common structural features. This alignment illustrates that U11 and U12 are the least well-defined residues in the model. An average structure was calculated, and the output structure with the smallest RMSD (1.31) from the average is shown in stereoview in Figure 8. This structure is taken to be the best representative of the ensemble. The average RMSD of the ensemble from the average structure is 2.53.

**Figure 9:**
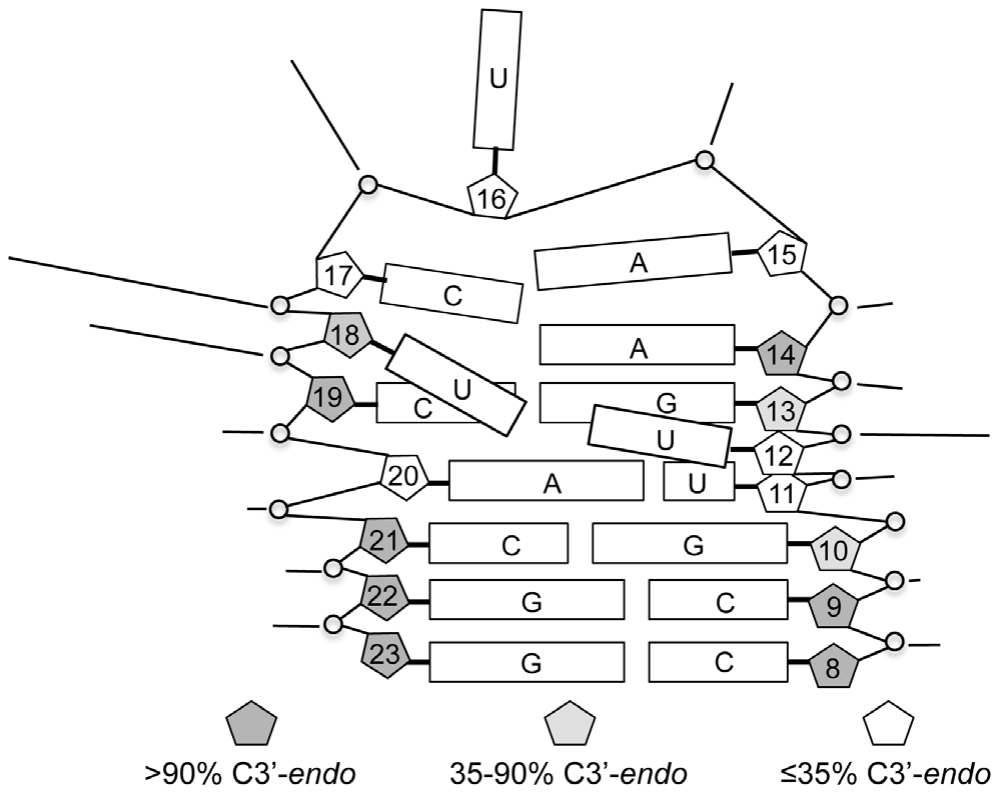
Schematic illustration of RNA 2 structure. Rectangles represent the bases of the indicated nucleotides. Base stacking is indicated by adjacent, parallel rectangles. Sugar pucker, as estimated from J_1′-2′_, is indicated by shading of pentagons representing the ribose ring for each nucleotide. Lines pointing to internucleotide linkages indicate relative intensity of Mg^2+^-induced cleavage at each position.

**Figure 10:**
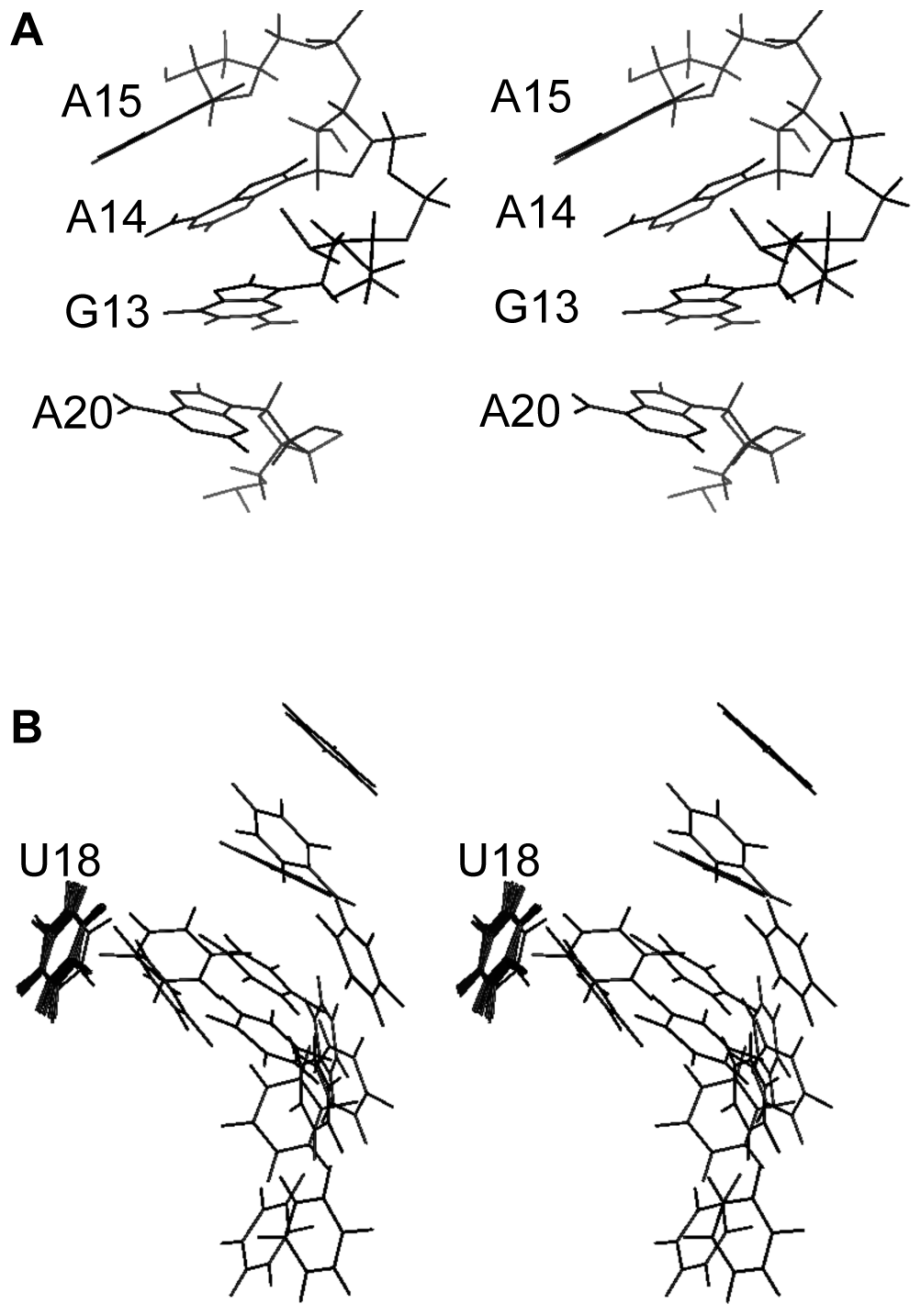
Stereoviews of structural features apparent in the NMR-based model of 2. A. Stacking of consecutive purines G13–A15 and cross-strand stacking of A20. B. Orientation of U12 with respect to U18. The fourteen lowest energy structures were aligned on U18 and the pyrimidine rings of U18 and U12 are shown.

The structure is shown schematically in Figure 9. In addition to stacking of sequential purines G13–A15, a sharp twist between the A20•U11 and C19•G13 base pairs places A20 under G13 in a cross-strand stacking interaction (Figure 10A). The loop turns at U16, and the pyrimidine ring is oriented out of the loop at this position. On the other hand, the pyrimidine ring of C17 is oriented toward the interior of the loop. U18 and U12 both protrude into the major groove, proximal to the edge of the A20•U11 base pair. Their distance and orientation with respect to each other are not well defined (Figure 10B); however, their Watson-Crick faces are generally oriented toward each other, and in several of the output structures they approach hydrogen bonding distance of each other. In a separate set of calculations, simulated annealing was carried out with these nucleotides restrained to within 2 Å (U18 H3–U11 O4) of each other. The lowest energy output structures had NOE and total energies equal to the lowest energies obtained without that constraint and shared the major conformational features of those structures.

## Discussion

The pre-element of miR-21 is an important subject of structural study because of its significance as a site of molecular recognition by the miRNA processing apparatus and because of its interesting predicted secondary structure. In addition to being important for recognition of pri-miR-21 by the microprocessor and pre-miR-21 by Dicer, it is a site of recognition by other endogenous factors that regulate interaction of the microprocessor with the primary transcript of miR-21 [31]. The strong sequence conservation of the apical loop, beyond what is required for recognition by factors that are known to bind this structure, suggests that there are still-undiscovered agents that form critical associations with the loop [27].

The predicted secondary structure of the loop, formally a five-nucleotide loop with a bulged nucleotide adjacent to the closing base pair, is unlike hairpin loops that have been studied previously by NMR [48]–[50]. Much of the structural analysis of hairpin loops has been directed to four-nucleotide loops such as the stable UNCG tetraloops [51] and the GNRA loop motif [52]. Several six-nucleotide hairpin loops have also been analyzed by solution methods [53]–[55]. However, there is relatively little structural information for five-nucleotide RNA loops, particularly in the context of a proximal bulge nucleotide as in the loop studied here.

The significance of the bulged uridine, U12, adjacent to the loop is underscored by its interesting and unexpected interaction with U18. Our NMR-derived model, in conjunction with the imino proton spectra of **2** and related hairpins, suggest a direct hydrogen bonding interaction between these nucleotides across the major groove. The orientation of U18 is similar to a feature seen in the solution structure of a five-nucleotide hairpin loop modeled after a loop in the 18S ribosomal RNA [48]. In that case, a cytosine at the 3′ end of the loop sequence is oriented toward the major groove of the adjacent double helix. That RNA, however, does not have an unpaired nucleotide available for interaction with the fifth loop nucleotide in the major groove. In contrast, the bulged uridine of **2**, also protruding into the major groove, provides an interaction partner for this nucleotide.

Molecular modeling suggests that the most likely hydrogen bonding partners for the interaction between U18 and U12 are U18 H3 and U12 O4. Such an interaction directs H3 of U12 inward toward the G13•C19 base pair, consistent with the partial protection of this proton from exchange with H_2_O. This arrangement also places the interacting uridines proximal to the major groove edge of the A20•U11 base pair, consistent with the loss or destabilization of the U12–U18 interaction upon alteration of this base pair. Though there is no direct evidence for a hydrogen bonding interaction between either of the interacting uridines and this A•U base pair, a hydrogen bond between U18 O2 and the exocyclic amine of A20 is a possibility.

### Relevance of the structure of RNA 2 to RNA 1 and the miR-21 pre-element

Whereas RNA **1** most closely models the miR-21 pre-element, RNA **2** provides a better system for structural study by NMR, because its secondary structure is more clearly defined by the observation of resonances due to imino protons. This added structural definition extended beyond the two predicted G•U base pairs that were changed in **2** to include observation of the imino protons of U5 and G6. It is important, however, to evaluate the relationship between the structures of **1** and **2**.

All of the peaks that are visible in the imino proton spectrum of **1** correspond to peaks in the spectrum of **2**. Those corresponding to G13 and G23, which are proximal to the altered nucleotides, have the same chemical shifts in both. Thus, the chemical environments of G13 and G23 are not dramatically altered between the two molecules. There is also a peak in the spectrum of **1** that corresponds to that assigned to U18 in **2**, though it is shifted downfield 0.2 ppm. The structural features that give rise to protection of this imino proton from exchange with H_2_O are apparently present in both RNAs. Reversion of only one of each of the altered base pairs to a G•U pair (RNAs **6** and **7**) results only in a loss of that G's imino proton from the spectrum and small changes in the chemical shift or intensity of peaks due to neighboring imino protons. These observations support the conclusion that **1** and **2** share most of their significant structural features.

The similarity of the in-line cleavage patterns for the two molecules further supports that conclusion. One of the primary differences in the patterns, stronger cleavage after U21 in **1** than after C21 in **2** can be attributed simply to lower stability of the base pairing in that stem. The other differences surround U12 and U18, which interact with each other. A perturbation in this interaction likely accounts for the alteration in backbone conformation or dynamics that alters the cleavage efficiency. The downfield shift of the imino proton peak for U18 between **2** and **1** is consistent with a perturbation of this interaction, but its presence in both suggests that the change does not entirely disrupt (or direct) the interaction but modifies its conformational details or dynamics. The lower stability of the duplex in **1** is sufficient to account for this modification. Thus, our data indicate that the conformation of RNA **2** resembles that of **1** in its most pronounced features.

### Relevance to maturation of miR-21

Zeng and co-workers have proposed a model in which the microprocessor and Dicer preferentially recognize conformations of the pre-element in which the base pairs are disrupted. They conclude that the predicted base paired regions as well as the loops of miRNA pre-elements are flexible [28], [29]. Our data for RNA **1** confirm the flexibility of the stem region of the miR-21 pre-element. Backbone conformations that allow in-line cleavage and sufficient breathing of the base pairs to allow exchange of imino protons with water are evidence of its dynamic nature.

The model proposed by Zeng and co-workers is supported by data for processing of pri-miR-21 mutants corresponding to RNAs **6** and **7**. Both of these mutations somewhat diminish cleavage by Drosha in vitro and maturation of pri-miRNA to the active miRNA in cultured cells [28], as predicted from the model in which disruption of base pairs in the pre-element aids recognition by the processing machinery. It is noteworthy, however, that both of these mutant pri-miRNAs are nonetheless processed appreciably [28], confirming that the structures of these mutant pri-miRNAs are relevant in a biological context. Furthermore, the imino proton spectra of **6** and **7** are essentially identical to that of **2**, except for the peaks due to G10 and G22, which are directly involved in the altered base pairs. This similarity establishes the structural similarity of **6** and **7** to **2** (as well as to **1**, as noted above). Thus, the structural features we have identified for **2** are consistent with processing of a pri-miRNA to functional maturity. Similarly, even a pri-miR-21 mutant with both G•U base pairs and the A•U base pair predicted in the pre-element converted to G•C base pairs (i.e., a mutant corresponding to **2** with the additional stabilization of U11•A20 converted to a G•C pair) is processed poorly but measurably [28].

The structural characteristics of **2** that we have determined are those of the isolated element of RNA secondary structure. Interactions with protein factors can refashion conformationally labile secondary structures. However, the high degree of conservation of the miR-21 pre-element suggests conservation of its distinctive conformation as well as its sequence. Thus, auxiliary factors may recognize this structure in the regulation of miR-21 processing. In the case of factors that do alter the conformation (i.e., bind and stabilize an altered conformation), the conformation of the free RNA provides a basis for probing and understanding the changes induced. It also provides a basis for characterization of the interaction of artificial ligands with pri-miR-21.

## Materials and Methods

### Sample preparation

RNA was obtained from ThermoFisher with 2′OH protective groups in place, deprotected according to vendor protocol, and precipitated with sodium acetate and ethanol.

### NMR Spectroscopy

Spectra of exchangeable protons were measured with the sample dissolved in a 10∶1 mixture of H_2_O and D_2_O containing 10 mM sodium phosphate, pH 6.7, and 50 µM EDTA. To measure spectra of non-exchangeable protons, the sample was dissolved in buffer and lyophilized to dryness before reconstituting in 600 µL of 99.96% D_2_O (Aldrich) to an RNA concentration of 1 mM. After reconstitution, the solution was heated briefly to 95°C and allowed to cool slowly to room temperature.

NMR experiments were recorded on Varian INOVA NMR spectrometers operating at proton frequencies of 600 and 800 MHz and equipped with cryogenic triple resonance probes. All experiments were acquired using standard pulse sequences from the library provided by the vendor's software. Spectra were processed using NMRPipe [56] and visualized and analyzed using NMRViewJ.

One-dimensional exchangeable proton spectra in H_2_O were collected at 5°C with a 1-1 water suppression sequence and a 1.2 s delay between pulses. A NOESY spectrum of the sample in H_2_O was acquired at 10°C (800 MHz spectrometer). Water suppression was achieved with a 1-1 pulse sequence having a 1.5 s delay between pulses. 160 scans were taken for each of 256 FIDs. The spectral width in both dimensions was 13587.0 Hz.

In all spectra of nonexchangeable protons, the residual HDO resonance was suppressed by presaturation during a 1.2 s relaxation delay. A double-quantum filtered COSY spectrum was acquired with a spectral width of 5421 Hz (600 MHz spectrometer), 16 scans, 1322 points in the directly detected dimension and 800 complex points in the indirect dimension. A series of NOESY experiments with 60 ms, 100 ms, 150 ms, and 400 ms mixing times was acquired on a 600 MHz spectrometer with 5421 Hz spectral width, 16 scans, 1322 FIDs, and 800 complex points. A NOESY spectrum with 400 ms mixing time was acquired on a 800 MHz spectrometer with 7227 Hz spectral width, 8 scans, 764 FIDs and 800 complex points. A natural abundance heteronuclear ^1^H-^13^C single quantum coherence (HSQC) spectrum was acquired (600 MHz spectrometer) using a standard pulse sequence and wurst140 for carbon decoupling during acquisition. The spectral width was 8012.8 Hz (13.4 ppm) in the ^1^H dimension and 16000 Hz (106 ppm) in the ^13^C dimension. 128 FIDs were collected with 2048 scans of 2728 complex points.

### In-line cleavage analysis

In-line cleavage, alkaline hydrolysis, and ribonuclease T1 digestion reactions were carried out and analyzed as previously described [42]. 5 mM MgCl_2_ was included in each in-line cleavage reaction.

### Structure Modeling

The three-dimensional structure of the pre-element (nucleotides 8–23 of RNA **2**) was modeled using a restrained molecular dynamics protocol incorporating NMR-derived distance and torsion angle restraints. Where a Watson-Crick base pair was indicated by the observation of an imino proton resonance, distance restraints were applied to maintain the appropriate hydrogen bonding distances and coplanarity of the bases. The distance between imino protons for which an NOE was observed was restrained to the range 2.0–4.5 Å.

NOE cross-peak intensities were used semiquantitatively to assign distance ranges to nonexchangeable protons. Crosspeak intensities were characterized as strong, medium, weak, and very weak according to the NOESY mixing times at which they were observable. Strong cross-peaks, observable with a mixing time of 60 ms, were assigned the range 1.8–3.0 Å. Medium-intensity cross-peaks, observable with a NOESY mixing time of 100 ms or longer, were assigned the range 2.0–4.0 Å. Weak cross-peaks, observable with mixing times of 150 ms or longer, were assigned the range 2.5–5.0 Å. Very weak cross-peaks were only seen in NOESY spectra with a 400 ms mixing time and were assigned the range 2.5–6.0 Å. Internucleotide distance constraints between C8 and C9 and between G22 and G23 were applied to model an A-form geometry for these residues, clearly indicated by presence of base pairing and internucleotide NOE intensities.

The sugar conformations were characterized by the H1′-H2′ scalar couplings evident in the DQF-COSY spectrum. Residues for which no H1′-H2′ coupling was observed were constrained to the C3′-*endo* conformation with the endocyclic torsion angles *ν*_0_, *ν*_1_, *ν*_2_, and *ν*_3_. Sugars with coupling ≥5.5 Hz were constrained to the C2′-*endo* conformation. Sugars with coupling <5.5 Hz were constrained to the range of conformations including C2′-*endo*, O4′-*endo*, and C3′-*endo*. Because all intranucleotide H1′ to aromatic NOESY crosspeaks were similar in intensity to those in the base paired lower stem (nucleotides 1–6 and 24–29), the glycosidic torsion angle, *Χ*, was constrained to the *anti* conformation for all nucleotides.

The molecular dynamics program CNS 1.3 was used to generate three-dimensional structures consistent with the NMR data. The covalent structure of the RNA was created in an extended conformation and subjected to a simulated annealing protocol, varying the initial velocities for multiple structure calculations. Torsion dynamics and the CNS default parameters for nucleic acids were used in each step. High temperature annealing was simulated with 4000 steps, 15 ps, at 36,000 K. Subsequently, 1000 steps of slow cooling were followed by 10 cycles of 200 final minimization steps. Structures were displayed using PyMol.
